# Prophylaxis of Patent Ductus Arteriosus with Paracetamol in Extremely Low Gestational Age Newborns (ELGANs): A Single-Institution Observational Study in Vietnam

**DOI:** 10.3390/children10121934

**Published:** 2023-12-17

**Authors:** Tinh Thu Nguyen, Dung Thi Ngoc Nguyen, Tam Thi Thanh Pham, Ju-Lee Oei

**Affiliations:** 1 Department of Pediatrics, University of Medicine and Pharmacy at Ho Chi Minh City, Ho Chi Minh City 700000, Vietnam; tinhnguyen@ump.edu.vn; 2 Neonatal Intensive Care Unit, Children’s Hospital 2, Ho Chi Minh City 700000, Vietnam; 3 Pediatric and Neonatology Department, Franco-Vietnamese Hospital, Ho Chi Minh City 700000, Vietnam; dung12.nguyen@fvhospital.com; 4 Neonatal Intensive Care Unit, Children’s Hospital 1, Ho Chi Minh City 700000, Vietnam; tamptt@nhidong.org.vn; 5 School of Women’s and Children’s Health, Faculty of Medicine, University of New South Wales, Sydney, NSW 2031, Australia; 6 Department of Newborn Care, The Royal Hospital for Women, Randwick, Sydney, NSW 2031, Australia

**Keywords:** patent ductus arteriosus, paracetamol, PDA closure, PDA ligation, respiratory distress syndrome

## Abstract

Introduction: Prophylactic paracetamol for extremely low gestation age neonates (ELGAN, <27 weeks’ gestation) with symptomatic patent ductus arteriosus (sPDA) in high-income countries (HIC) reduces medical and surgical interventions. Its effectiveness in low-to-middle-income countries (LMIC) remains uncertain. This study assesses prophylactic paracetamol’s impact on sPDA interventions in ELGANs in an LMIC. Methods: This is a retrospective cohort study that compared a historical cohort of ELGANs that were treated with oral ibuprofen or intravenous paracetamol after diagnosis of sPDA (*n* = 104) with infants (*n* = 76) treated with prophylactic paracetamol (20 mg/kg loading, 7.5 mg/kg qid for 4 days), in a tertiary neonatal intensive care unit (NICU) in Vietnam. Oral ibuprofen or intravenous therapeutic paracetamol were administered if prophylactic paracetamol failed to close sPDA. Surgical ligation was conducted if targeted medical intervention failed, or the infant deteriorated from conditions attributable to sPDA. Results: In the historical cohort, 57 (55%) infants died within 7 days of life compared to 18 (24%) from the prophylactic cohort (*p* < 0.01). Of the survivors, 21 (45%) of the historical and 23 (39.7%) of the prophylactic cohort required surgical ligation (*p* = 0.6). Duration of hospitalization for survivors was lower in the prophylactic cohort (mean 74 vs. 97 days, *p* = 0.01). In the prophylactic cohort, 24 (41%) infants did not need further treatment while 34 (59%) required further treatment including ibuprofen and/or paracetamol 28 (48%) and surgical ligation 22 (38%). Conclusions: Prophylactic paracetamol for ELGAN in LMIC does not reduce the need for surgical ligation, sPDA rates, and other PDA-related morbidities in infants who survive beyond 7 days of age. It may reduce the risk of death and the duration of hospitalization but further study into the reasons behind this need to be determined with larger studies.

## 1. Introduction

Patent ductus arteriosus (PDA) is a common problem among extremely low gestation (ELGAN, below 29 weeks’ gestation age), affecting 72.4% of infants by the end of the first week of life [1]. Of these, 57% have hemodynamically significant PDAs (hsPDA), which may compromise circulatory and respiratory function [1,2,3]. Untreated hsPDA may worsen respiratory distress [4] and increase the risk of severe intraventricular hemorrhage (IVH) and pulmonary hemorrhage, which usually occurs within 72 h after birth and affects mortality and long-term neurological development [3,5].

Unfortunately, detection of hsPDA is difficult, especially when resources, such as the expertise to conduct echocardiograms, are limited. In high-income countries (HIC) with advanced medical support, prophylactic treatment of ELGANs with IV paracetamol has been shown to reduce the frequency of hsPDA and the need for medical and surgical intervention [5,6]. However, low-and-middle-income countries (LMIC) have differing levels of resources and staff expertise and results of research from HIC may not translate into benefits for ELGANs from LMIC.

In Vietnam, a LMIC in Indochina, the current treatment of hsPDA (i.e., low diastolic pressure, fluid overload, worsening respiratory distress) involves targeted closure with non-steroidal anti-inflammatory drugs (NSAIDs), mainly with oral ibuprofen. Surgical ligation of persistent and symptomatic PDAs is considered a last resort for infants who fail medical treatment or who have a contraindication to NSAID treatment (e.g., bleeding or renal disorders). Surgical treatment is only available in two hospitals in Vietnam, creating significant logistic and financial difficulties for the parents if an infant needs ligation. There are also no facilities or expertise for catheter closure of PDAs in ELGANS in Vietnam.

The largest Newborn Intensive Care Unit (NICU) in Vietnam is the Children’s Hospital 1 (NICU CH1) in Ho Chi Minh City. This is a level IV NICU that primarily manages outborn infants transferred from other newborn and maternity units in the area surrounding Ho Chi Minh City. The prevalence of PDA and hsPDA among premature infants born before 28 weeks gestational with respiratory distress syndrome at the end of the first week in this hospital is 90% and 70%, respectively [7]. Due to difficulties in timely diagnosis and treatment, pulmonary hemorrhage from hsPDA remains one of the most common causes of death in infants < 27 weeks gestational age [8].

Since 2018, our NICU has used prophylactic IV paracetamol in ELGANs in an effort to reduce side effects from hsPDA. However, whether this has decreased adverse outcomes, including pulmonary hemorrhage, bronchopulmonary dysplasia (BPD), and death, as well as the need for further PDA intervention, is unclear. In this retrospective study, we hypothesized that the use of prophylactic IV paracetamol in ELGANs will decrease the risk of hsPDA development, the need for further PDA intervention, and morbidities including bronchopulmonary dysplasia (BPD) and death.

## 2. Methods

This single-institution observational study was conducted at the NICU of Children’s Hospital 1 in Ho Chi Minh City, Vietnam. Infants were included in this study if they were admitted to the NICU from April 2018 to February 2021 and if they fulfilled the following criteria—(i) prematurity ≤ 27^0/7^ weeks GA; (ii) respiratory distress syndrome (RDS); (iii) receipt of IV paracetamol within the first 24 h after birth—according to the following regimen: 20 mg/kg loading dose, and then 7.5 mg/kg every 6 h for 4 days. Exclusion criteria included (i) major congenital abnormalities incompatible with life; and/or (ii) failure to complete the course of paracetamol prophylaxis; and/or (iii) failure to receive an echocardiography after completion of paracetamol prophylaxis; and/or (iv) death before diagnosis of PDA. We used poractant alfa 80 mg/mL for surfactant with an initial dose of 200 mg/kg that was repeated once after 6 h if fractional inspired oxygen (FiO_2_) exceeds ≥ 0.3.

## 3. Comparison Cohort

The outcomes of this cohort were compared to a historical group of ELGANs who were admitted to the NICU between October 2017 and December 2019. This cohort received the same standard treatment in the NICU except for paracetamol prophylaxis (see Appendix A and Appendix B). This cohort was also only treated with either paracetamol or ibuprofen when a hsPDA was diagnosed.

Primary outcomes were (i) the incidence of hsPDA after completion of the prophylactic paracetamol course; and (ii) the need for medical and surgical intervention.

Secondary outcomes were (i) need inotropic agents to treat hypotension within the first three days of life; (ii) pulmonary hemorrhage (PH); (iii) peri/intra-ventricular hemorrhage (P/IVH); (iv) necrotizing enterocolitis (NEC) > Bells grade 2; (v) acute renal failure (ARF); (vi) bronchopulmonary dysplasia (BPD), defined as the need for supplemental oxygen and/or respiratory support at or after 36 weeks postmenstrual age; (vii) retinopathy of prematurity (ROP) requiring surgery; (viii) duration of respiratory support; (ix) duration of hospitalization; and (x) survival to hospital discharge.

## 4. Definitions

(1) hsPDA was defined as a PDA diameter/birth weight (kg) of ≥ 1.4 (mm/kg) and one of the additional echocardiographic findings: LA/Ao > 1.4 and/or PDA diameter/LPA (left pulmonary artery) diameter > 0.5 [9,10]. Ductus that failed to meet these criteria were non-hsPDA. No PDA was defined as no flow through the ductus.
(2) sPDA (symptomatic PDA) was defined as the presence of hsPDA and one of the following conditions attributable to the hsPDA, including the following [2,3]: (1) the need for vasopressor support; (2) a persistent or increasing need for ventilatory support and supplemental oxygen; (3) prerenal renal failure with metabolic acidosis; (4) intraventricular hemorrhage (IVH) Papile’s grade of ≥II [11] on ultrasound; (5) renal failure, defined as serum creatinine that is >1.5 mg/dL and/or serum creatinine rise of >0.3 mg/dL or serum creatinine rise of >1.5–1.9* the lowest previous serum creatinine value [12]; (6) necrotizing enterocolitis (NEC) ≥ Bell’s stage 2 or greater [13]; and (7) pulmonary hemorrhage. These were defined by meeting all three of following criteria: (1) fresh blood in the trachea or the endotracheal; (2) a rapid clinical deterioration need for intubation or increase FiO_2_ ≥ 10% for children on mechanical ventilation, decreased hematocrit > 10%; and (3) chest radiograph—patchy infiltrates or complete opacification).

## 5. Clinical Management

In the prophylactic group, IV paracetamol was commenced within 6 h of life (further detail in Appendix A and Appendix B). In the historical group, paracetamol or ibuprofen was administered if the infant was symptomatic from a PDA. For the prophylactic group, no further treatment was provided if the ductus was closed, or the PDA was asymptomatic on a screening echocardiogram conducted after the initial course of paracetamol or at any time if the infant was symptomatic. All infants in the prophylactic group were examined daily, or, more often, with cardiac ultrasounds if clinical signs indicative of a sPDA (systolic murmur, widened pulse pressure, and hyperdynamic precordium) developed. Cardiac ultrasounds were performed by a pediatric cardiologist who was blinded to the interventions above.

In both groups, infants with sPDA who failed the first course of paracetamol were treated with oral ibuprofen 10 mg/kg/dose every 24 h for 3 doses if there were no contraindications. Intravenous (IV) paracetamol 15 mg/kg/dose every 6 h for 3 days was used if ibuprofen was contraindicated or if ibuprofen failed. Surgical ligation was conducted when these drugs were contraindicated, had failed or if the infant deteriorated clinically in the context of a hsPDA.

Contraindications for oral ibuprofen treatment included oral feeding intolerance, suspected or confirmed NEC, severe sepsis, active bleeding (new mucosal hemorrhages, IVH ≥ grade 2), thrombocytopenia < 60,000/mm^3^, kidney function disorders (oliguria < 0.5 mL/kg/h within 8 h, serum creatinine > 1.5 mg/dL), and severe hyperbilirubinemia (total serum bilirubin level is higher than the exchange level according to gestational age and postnatal age) [2]. Contraindications for IV paracetamol were abnormal liver function tests, including AST > 150 U/L or ALT > 90 UI/L.

### Statistical Analyses

Data were analyzed using SPSS statistics, version 20. The primary outcome of the need for medical treatment or surgical ligation was analyzed for patients who survived the first week of life after completion of the prophylactic paracetamol course. Infants who failed to complete the prophylactic paracetamol course (e.g., death, other complications) were excluded from the analysis of the primary outcome. Analyses for other outcomes were conducted on an intention-to-treat basis. Shapiro–Wilks statistics were used to determine the distribution of continuous variables, which were examined by the Student’s *t*-test for normally distributed values and the Mann–Whitney U test for non-normally distributed values. Categorical variables were compared by using the Chi-square test. The level of statistical significance was set at *p* = 0.05 for all tests.

## 6. Results

### 6.1. Patient Characteristics

**Figure 1:**
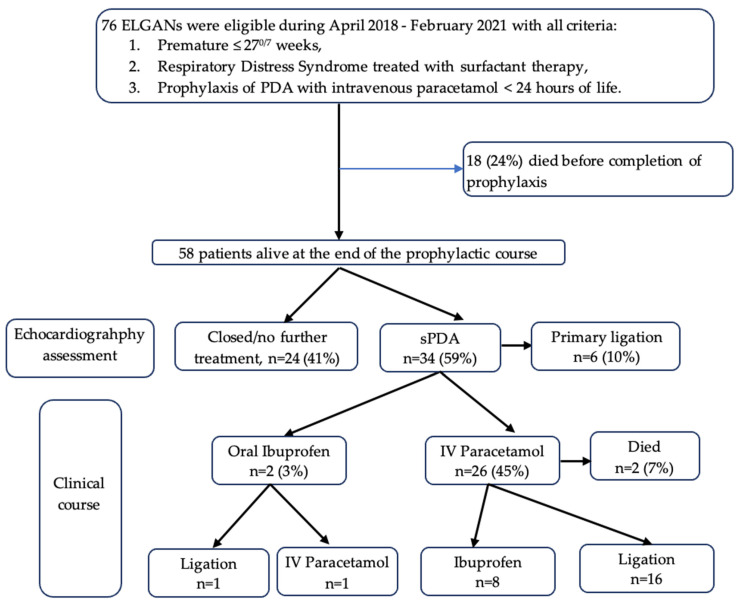
Flowchart of the study. ELGAN (extremely low gestational age newborn), PDA (Patent Ductus Arteriosus), sPDA (Symptomatic Patent Ductus Arteriosus).

The historical control consisted of 104 ELGANS admitted between October 2017 and December 2019. Fifty-seven (55%) infants died within the first week of life (pulmonary hemorrhage (34, 60%), septic shock (13, 23%), pneumothorax (4, 7%), and multiple/undetermined etiologies (6, 10%). The baseline characteristics are detailed in Table 1. In the prophylactic cohort, 76 infants were included between April 2018 and February 2021. Eighteen (24%) infants died before completion of the paracetamol course (Figure 1). A total of 58 patients were included for analysis of the co-primary outcome (Table 1).

### 6.2. Prophylactic Paracetamol Treatment

In the prophylactic cohort, IV paracetamol was started at a median of 6 h postnatal age. Cardiac ultrasounds were performed at median day 5 of life after completion of the prophylactic course. The rates of closed PDA, no sPDA, and sPDA were 16 (28%), 8 (14%), and 34 (58%), respectively. The clinical characteristics of the groups that had or did not have a sPDA after the end of the prophylactic course were not different (Table 2).

### 6.3. Outcomes of Prophylactic Paracetamol Treatment

In the prophylactic cohort, 16 (28%) patients were not given further PDA treatment. Thirty-four patients needed further intervention. One infant required emergency ligation because of severe heart failure associated with progressive NEC whilst undergoing paracetamol therapy. Five others developed symptomatic PDA after 2 weeks of age and underwent ligation. Two (3%) received oral ibuprofen and 26 (45%) patients received IV paracetamol as ibuprofen was contraindicated. Two of twenty-six infants died before completion of the therapeutic paracetamol course. Among the other 32 cases of sPDA, the rate of successful PDA closure by oral ibuprofen and paracetamol IV were 1/2 (50%) and 8/26 (31%), respectively. Surgical ligation was required in 23/32 (72%). Overall, the surgical ligation rate was 29/58 (50%).

### 6.4. Morbidities and Mortality

These are detailed in Table 3. The risk of death prior to 7 days of age was higher in the historical cohort (55% and 24%, respectively, odds ratio (OR) of 3.9, 95% confidence interval (CI) 2.03–7.52), *p* < 0.01). Nine patients died before 28 days of age and eight patients died after 36 weeks postmenstrual age. Among the 49 infants who survived to 36 weeks’ gestation, 46 (94%) were diagnosed with BPD. Twenty (35%) had moderate and severe BPD. Among 44 patients alive over 31 weeks of age, 12 (27%) were treated with laser or Avastin for ROP > stage 3.

## 7. Discussion

This paper describes the use of prophylactic paracetamol in a cohort of ELGANs in a LMIC. Closure of a sPDA can be achieved through medical or surgical interventions. Medical approaches encompass the use of non-steroidal anti-inflammatory drugs like ibuprofen or indomethacin, as well as acetaminophen. Surgical interventions involve procedures such as surgical ligation and transcatheter occlusion [3]. Our results indicate that there is potential benefit for prophylactic paracetamol treatment when guided by echocardiogram, to decrease the need for surgical and medical intervention in hsPDA in this high-risk population. This is important because the risks and morbidities of hsPDA in ELGANs are very different to infants in HIC, due to differences in availability of resources including surgical expertise and other treatment interventions.

Firstly, the use of antenatal steroids was low, at 28%. Antenatal steroids reduce the risk of significant PDA in preterm infants by promoting ductus arteriosus constriction via PGE_2_ breakdown, decreasing the PGE_2_ sensibility of the ductus arteriosus, and possibly enhancing ion channel expression and/or reducing the incidence of RDS [14]. Although the aim of primary obstetric care in LMIC is to increase the uptake of preventative strategies such as antenatal steroids to reduce infant morbidity and mortality, strategies to address the consequences of sub-optimally prepared preterm births are needed in the interim.

Secondly, access to interventions that reduce the severity of respiratory illness such as timely receipt of surfactant, is lower in LMIC than in HIC. For example, 65.6% of patients had a 5 min APGAR score ≤ 6, and one-third required intubation resuscitation in the delivery room. None could be given surfactant until their admission to our NICU, a median of more than 6 h from birth, which is also associated with an increase in the risk of hsPDA [15].

Thirdly, respiratory support during transportation to the NICU of Children’s Hospital 1 or in the birth hospital may be inadequate which again, may increase the risk of hsPDA. For example, many obstetric hospitals in LMIC do not have appropriate equipment for appropriate respiratory support (e.g., PEEP). In our cohort, infants were transferred with an oxygen cannula (10.3%) or bag-mask ventilation via an endotracheal tube (43.1%) without access to humidified gas. Severe respiratory failure can affect the vasoconstrictive response of high oxygen tension in ductus arteriosus and ineffective limitation of PGE2 through the lungs [14].

Fourthly, a majority of ELGANs in both the prophylactic paracetamol cohort and historical control in our study had hypothermia on admission. A meta-analysis of eighteen studies on outcomes of neonatal hypothermia among very low birth weight (VLBW) infants showed a high rate of neonatal hypothermia, and it is a risk factor for mortality and morbidity in this population. Hypothermia on admission was significantly associated with adverse outcomes, including mortality (OR = 1.89; 95% CI: 1.72–2.09), intra-ventricular hemorrhage (OR = 1.86; 95% CI: 1.09–3.14), bronchopulmonary dysplasia (OR = 1.28; 95% CI: 1.16–1.40), neonatal sepsis (OR = 1.47; 95% CI: 1.09–2.49), and retinopathy of prematurity (OR = 1.45; 95% CI: 1.28–1.72) [16].

In our study, after the introduction of prophylactic IV paracetamol, the rate of sPDA at 5 days of life decreased from 70% to 59%. However, despite this reduction, the rate of symptomatic PDA requiring interventions remained high at 34 out of 58 (59%) cases. The efficacy of pharmacological treatments (oral ibuprofen and paracetamol) in closing PDA was only 9 out of 34 (27%) cases. Surgical ligation, as a backup after the pharmacological intervention, was in 23 out of 34 (68%) cases. In our population, surgical ligation of symptomatic PDA was backup for one course of oral ibuprofen or IV paracetamol (only one patient received two courses: paracetamol and then oral ibuprofen). Roofthooft et al. reported a surgical ligation rate for hsPDA after the failure of IV ibuprofen and/or IV paracetamol of 76.6% [17]. This is a higher rate than our study but may be due to institutional variation and access to surgical expertise. While some studies have shown that prophylactic intravenous paracetamol decreases the need for surgical ligation in extremely preterm infants [2,18], our findings did not align with these outcomes. It indicates that there might be variability in the efficacy of prophylactic intravenous paracetamol across different populations between HIC and LIMC. The 7-day mortality decreased from 55% to 24% (*p* < 0.01). Additionally, late deaths, occurring beyond 7 days of life, were higher in the paracetamol cohort (22 out of 58, 38%) compared to the historical control (28 out of 47, 60%) (*p* = 0.03). Furthermore, the total deaths upon discharge were higher in the paracetamol cohort (40 out of 76, 52.6%) compared to the historical control (85 out of 104, 82%) (*p* = 0.00003). However, some biases might have impacted the differences.

Pulmonary complications remained a significant concern in our study cohort, contributing to a substantial portion of mortality cases (in 10 out of 18 cases, 56%) within the first week of life. Among fifty-eight patients analyzed, pulmonary hemorrhage was 18 cases (31%) and BPD was diagnosed in 93.9%. A large-scale randomized controlled trial would be needed to examine the impact of prophylactic paracetamol on these outcomes.

Although the rate of sPDA on 5 days of life in historical control (70%) was higher than in the prophylactic cohort (59%), this was not statistically significant due to the small sample size and raises the need for us to address limitations in our study. The use of a mix of retrospectively and prospectively collected data introduced challenges in controlling various factors, including the timing of echocardiograms, different clinician decisions (e.g., fluid volumes, ventilation strategies), and the potential impact of referring hospital management on the infant’s cardiorespiratory outcomes. Furthermore, the lack of blinding during data collection could have introduced biases that might have impacted our results, and the use of historical controls raises questions about the potential confounding effect of changes in clinical practice over the study period, which had been over five years, from 2017 to 2021 (see Appendix A and Appendix B).

Nevertheless, our study provides evidence that using prophylactic intravenous paracetamol has the potential to reduce mortality and length of hospital stay in ELGANs in an LMIC setting. However, we have not shown a reduction in the incidence of sPDA, the need for surgical ligation, and other PDA-related morbidities. This is important in settings where patients may not be able to access appropriate antenatal or early postnatal care or diagnostic and interventional expertise such as echocardiography and surgical ligation. A large-scale, prospective RCT should be conducted to evaluate the effect of prophylactic intravenous paracetamol on this problem in LMIC.

## Figures and Tables

**Table 1:** Baseline characteristics of patients alive by day 7.

Parameters	Prophylactic *n* = 58	Historical * n = 47	*p*-Value
Gender, male	20 (34.5)	22 (46.8)	0.20
Gestational age (weeks) (min–max)	25.1 ± 1.1 (23^+0/7^–27^+0/7^)	26 ± 0.9 (24^+0/7^–27^+0/7^)	0.29
Birth weight (g) (min–max)	766.7 ± 183.5 (400–1300)	871.2 ± 134.3 (500–1400)	0.19
Cesarian section	5 (8.6)	4 (8.5)	0.98
Multiple births	28 (48.3)	19 (40.4)	0.42
PROM ≥ 18 h	7 (12.1)	1 (2.1)	0.06
Antental steroids	16 (27.6)	12 (25.5)	0.81
5 min APGAR score ≤ 6	38 (65.6)	29 (61.7)	0.69
Delivery room resuscitation:			
○ Oxygen cannula;	7 (12.1)	5 (10.6)	0.84
○ Nasal CPAP;	32 (55.2)	24 (51.1)	
○ Intubation.	19 (32,7)	18 (38.3)	
Surfactant administration in the delivery room	0 (0.0)	0 (0.0)	-
Respiratory support during transportation:			
○ Oxygen cannula;	6 (10.3)	2 (4.3)	0.50
○ Nasal CPAP;	27 (46.6)	23 (48.9)	
○ Intubation.	25 (43.1)	22 (46.8)	
Temperature at admission (°C)	33.6 ± 1.8	34.2 ± 1.2	0.43
MV within the first 24 h	51 (87.9)	38 (80.9)	0.32
Surfactant administration:			
○ 1 dose;	29 (50)	20 (42.6)	0.45
○ ≥2 doses.	29 (50)	17 (36.2)	0.16
Age at start surfactant (h)	6 (4–8)	6 (4–9)	0.75
Age at start IV paracetamol (h)	9 (6–20)	10 (4–21)	0.58

* Historical infants received paracetamol or ibuprofen only for symptomatic treatment of PDA. Abbreviations, PROM: premature rupture of membranes, MV: mechanical ventilation.

**Table 2:** Comparison of baseline characteristics of sPDA and no sPDA groups in the prophylactic paracetamol group.

Parameters	sPDA		*p*-Value
	No *n* = 24	Yes *n* = 34	
Gender, male	9 (37.5)	11 (32.4)	0.68
GA (weeks)	25.1 ± 1.1	25.1 ± 1.1	0.71
BW (g)	768.1 ± 185.1	765.7 ± 185.1	0.78
SGA	1 (4.2)	2 (5.9)	0.63
Cesarian delivery	2 (8.3)	3 (8.8)	0.66
Multiple pregnancies	15 (62.5)	13 (38.2)	0.07
PROM ≥ 18 h	2 (8.3)	5 (14.7)	0.72
Chorioamnionitis	1 (4.2)	2 (5.9)	0.96
No antenatal steroids	16 (66.7)	20 (58.8)	0.54
5 min APGAR score ≤ 6	15 (68.2)	23 (79.3)	0.36
Resuscitation via ETT	7 (29.2)	12 (35.3)	0.62
Temperature at admission	33.7 ± 1.7	33.5 ± 1.8	0.57
MV within the first 24 h	20 (83.3)	31 (91.2)	0.43
Age at start surfactant (h)	6 (4–7)	6 (4–8)	0.79
Age at start IV paracetamol (h)	8 (6–18)	10 (5–20)	0.69

Abbreviations. GA: gestational age, BW: birth weight, SGA: small for gestational age, PROM: premature rupture of membranes, ETT: endotracheal tube, MV: mechanical ventilation.

**Table 3:** Outcomes of infants in the prophylactic and historical cohorts alive after day 7.

Outcome	Prophylactic *n* = 58	Historical * n = 47	*p*-Value
sPDA on 5 days of life	34 (58.6)	33 (70.2)	0.22
Hypotension requiring vasopressor support within the first 3 days of life	24 (41.4)	19 (40.4)	0.92
Pulmonary hemorrhage	18 (31.0)	17 (36.2)	0.58
Peri/intraventricular hemorrhage	28 (48.3)	24 (51.0)	0.78
Peri/intraventricular hemorrhage, Grade ≥ 2	10 (17.2)	16 (34.0)	0.05
Necrotizing enterocolitis, Grade ≥ 2	6 (10.3)	9 (19.1)	0.20
Renal failure	9 (15.5)	Not available	-
BPD (grade moderate to severe)	20 (34.5)	24 (51.1)	0.09
ROP was treated with laser or Avastin	12/44 (27.3)	7/36 (19.4)	0.41
Respiratory support duration (days)	62 ± 38	64 ± 36	0.71
Invasive mechanical ventilation duration (days)	36 ± 30	21 ± 25	0.20
Hospital stay (day)	74 ± 40	97 ± 28	0.01

* Historical infants received ibuprofen or paracetamol only for symptomatic PDA. Abbreviations: BPD: Bronchopulmonary Dysplasia, sPDA: Symptomatic Patent Ductus Arteriosus.

## Data Availability

The data presented in this study are available on request from the corresponding author. Data contained within this article are not available due to privacy issues.

## Appendix A. Prevention and Management Protocol for Patent Ductus Arteriosus in Preterm Infants below 35 Weeks’ Gestational Age at Our Unit

I. Overview

The patent ductus arteriosus (PDA) is a fetal physiological structure that assists fetal survival; it normally closes after birth. The proportion of PDA in preterm infants at 3 days of age is around 60%, increasing with lower gestational age. PDA that does not close after birth can impact the heart and target organs, and increase mortality.

II. Diagnosis

1. Clinical symptoms: often asymptomatic.

- Cardiac symptoms: Hyperdynamic precordial impulse, precordial systolic murmur, tachycardia, bounding pulses, widened pulse pressure, hypotension, cardiac failure, inability to discontinue mechanical ventilation without explained causes. Congestive heart failure usually appears after one week of age: cardiomegaly, pulmonary congestion, and hepatomegaly.
- Organ effects: Lungs (worsening respiratory status, increased pulmonary blood flow, pulmonary edema, pulmonary hemorrhage), gastrointestinal tract (poor feeding, necrotizing enterocolitis), kidneys (oliguria, increased blood creatinine), brain (intraventricular hemorrhage).
- PDA reopening: Worsening clinical status with increased respiratory support needs, significant apnea, inability to wean from mechanical ventilation, unexplained kidney failure, poor feeding or necrotizing enterocolitis, and new onset intraventricular hemorrhage.

2. Work-up:

- Echocardiography:

- Screening: performed between 48 and 72 h for preterm infants.
- Evaluation: whenever there are clinical symptoms.
- Goals:
  ✓ Exclude associated heart defects: PDA-dependent critical CHDs (especially coarctation), pulmonary stenosis.
  ✓ Assess PDA diameter, ductal flow pattern, left atrium to aorta ratio (LA/Ao), and presence of reversed diastolic flow in the Superior Mesenteric Artery, descending aorta, or middle cranial artery.

- Chest X-ray: heart size, lung fields, associated injuries.
- Lab tests before pharmaceutical PDA closure:

- For ibuprofen: blood test, renal function, stool examination, cranial ultrasonography, and abdominal ultrasound;
- For Paracetamol: AST and ALT.

- Preoperative tests for preparing PDA ligation: ECG, complete blood count, renal function, electrolyte panel, and liver function.

3. Diagnosis: determined through echocardiography, and in case of surgical PDA ligation, results from at least two experienced cardiologists are necessary.

Symptomatic PDA (sPDA) as a combination of PDA diameter/weight ≥ 1.4 (mm/kg) with left-to-right PDA shunt on echocardiography along with one of the following (clinical signs of a high-volume left-to-right shunt):

✓ Hyperdynamic precordial impulse.
✓ Tachycardia without explained causes.
✓ Widened pulse pressure (Diastolic Blood Pressure (DBP) < ½ Systolic Blood Pressure (SBP)).
✓ Left atrium diameter to aortic diameter ratio (LA/Ao) > 1.4.
✓ Reversed diastolic flows in the superior mesenteric artery, descending aorta, or middle cerebral artery.
✓ Increased respiratory support needs or inability to discontinue mechanical ventilation without explained causes.
✓ Kidney failure with metabolic acidosis without explained causes.
✓ Progressive necrotizing enterocolitis grade ≥ 2 and/or new onset of intraventricular hemorrhage grade ≥ 2.

III. Treatment

Treatment goals: minimize the hemodynamic impact of PDA on the heart, lungs, gastrointestinal tract, kidneys, and brain.

1. Prevention of sPDA

- Indications: preterm birth ≤ 27 weeks; respiratory distress syndrome requiring surfactant therapy;
- Timing: within the first 24 h after birth;
- Dosage: IV Paracetamol, loading dose 20 mg/kg, followed by 7.5 mg/kg every 6 h for 4 days.

2 Management of PDA

a. Conservative management in the first week of life for all infants with respiratory distress:

Maintain thermal stability, restrict intake fluids (keep fluid balance is 0 or negative provided meets nutrition requirement), prevent anemia (Hct 35–40%), target SpO_2_ 92–95%, avoid loop diuretics, NCPAP with pressure (P) ≥ 5 cmH_2_O, and mechanical ventilation with Inspiratory Time (Ti) < 0.35″.

b. Medical intervention:

Indications: symptomatic PDA.

Oral ibuprofen contraindications: if there is minimal milk intake or necrotizing enterocolitis is being monitored; progressing septicemia; evolving bleeding (petechiae, bloody stools, intraventricular hemorrhage of ≥ grade 2); platelet count < 60,000/mm^3^; significant renal dysfunction (urine output < 0.5 mL/kg/hour for 8 h or Creatinine > 1.5 mg/dL); significant jaundice with indirect bilirubin approaching exchange threshold.

Paracetamol intravenous contraindication: AST > 150 U/L or ALT > 90 U/L.

c. Surgical intervention (PDA ligation):
  - sPDA with contraindications to or failure of medical intervention;
  - sPDA-related severe respiratory failure, progressive NEC grade II–III, or new onset of IVH grade III–IV.

IV. Monitoring

- Monitor for PDA reopening (see clinical symptoms above).

**Recommendations**	**GRADE**
Conservative management for all preterm infants with respiratory distress. If diuretics are needed, thiazides are preferable to loop diuretics.	1B
Conservative management for all preterm infants with respiratory distress. If diuretics are needed, thiazides are preferable to loop diuretics.	1B
Ibuprofen preferable over Indomethacin for PDA closure.	1B
In preterm infants with respiratory distress and mechanical ventilation lasting > 1 week, drug closure is preferred over conservative management.	2B
Surgical PDA ligation indicated in infants with symptomatic PDA, high ventilation settings, and pharmaceutical intervention failure.	2B
Indomethacin not recommended for prophylaxis to reduce PDA in preterm infants.	1B
Paracetamol recommended for prophylaxis in infants ≤ 32 weeks to reduce PDA occurrence.	2C
Note: Strength of recommendation. 1: strong recommendation, 2: weak recommendation. Level of evidence—A: high quality, B: moderate quality, C: low quality.	
